# Reply to: Caution in inferring viral strategies from abundance correlations in marine metagenomes

**DOI:** 10.1038/s41467-018-08286-4

**Published:** 2019-01-30

**Authors:** F. H. Coutinho, C. B. Silveira, G. B. Gregoracci, C. C. Thompson, R. A. Edwards, C. P. D. Brussaard, B. E. Dutilh, F. L. Thompson

**Affiliations:** 1 Universidade Federal do Rio de Janeiro (UFRJ), Instituto de Biologia (IB), Rio de Janeiro, 21944970 Brazil; 2 Radboud University Medical Centre/Radboud Institute for Molecular Life Sciences, Centre for Molecular and Biomolecular Informatics (CMBI), Nijmegen, 6500 HB The Netherlands; 3 Theoretical Biology and Bioinformatics, Utrecht University (UU), Utrecht, 3584 CH The Netherlands; 4 Biology Department, San Diego State University (SDSU), San Diego, 92182 USA; 5 Departamento de Ciências do Mar, Universidade Federal de São Paulo (UNIFESP), Baixada Santista, 11070100 Brazil; 6 Department of Biological Oceanography, Royal Netherlands Institute for Sea Research (NIOZ), Texel, 1790 AB The Netherlands; 7 Department of Aquatic Microbiology, University of Amsterdam/Institute for Biodiversity and Ecosystem Dynamics (IBED), Amsterdam, 1090 GE The Netherlands; 8 Universidade Federal do Rio de Janeiro (UFRJ)/COPPE/SAGE, Rio de Janeiro, 21941950 Brazil

**Replying to** H. Al-Rasheed et al. *Nature Communications* 10.1038/s41467-018-07950-z (2019)

Our publication by Coutinho et al.^1^ assembled viral genomes from marine viromes and linked these viral genomes to their potential hosts. We then investigated the relationship between the abundances of microbes (hosts) and virus-host-ratios (VHR) across samples (see Fig. 6 in ref. ^1^). We observed a decrease in VHR with increasing host abundance that is consistent with independent findings^2,3^. That result was interpreted in the discussion of our original publication as a relative decrease in lytic viral production. The decrease in VHR with increasing host abundance is consistent with the Piggyback-the-Winner (PtW) model, which proposes that lysogeny is the mechanism behind the decrease in VHR^4^. Al-Rasheed et al.^5^ criticized our interpretation arguing that the negative relationship reported for VHR and host abundance was not indicative of higher frequency of lysogeny.

This criticism seems to stem from the interpretation of PtW. In Coutinho et al.^1^, we refer to PtW as a framework that proposes an increase in lysogeny at high host abundances, resulting in the decrease in VHR. Al-Rasheed et al.^5^ refer to the specific mathematical formulation presented in Fig. 1B of Knowles et al^4^. In Coutinho et al.^1^, we analyzed how the ratio between viral and bacterial abundances (*y/x*) responded to bacterial abundances following a power function *y/x* ~ *x*^*β*^. This test led to *β* ≈ –1. Al-Rasheed et al. pointed out that this result masked the relationship between viral and bacterial abundances, *y* ~ *x*^*α*^ which resulted in a null slope *α* ≈ 0 for most virus-host pairs (see Fig. 1a in Al-Rasheed et al.^5^). Because this relationship does not match that of the mathematical formulation in Fig. 1B of Knowles et al.^4^, Al-Rasheed et al.^5^ argued that there was no evidence to support PtW in our data. However, we do not restrict our interpretation to the mathematical formulation in Fig. 1B of Knowles et al.^4^ because that is an extension of the Kill-the-Winner formulation modified so the lytic production decreases with increasing microbial abundances, with no explicit lysogenic component.

The results of both analyses (*y* ~ *x*^*α*^ by Al-Rasheed et al.^5^ and *y/x* ~ *x*^*β*^ by Coutinho et al.^1^) show that the viral abundance did not proportionally increase upon increase in host abundance. That is interpreted here, in agreement with previous literature, as a relative decrease in lytic activity^6,7^. The rationale is that high bacterial and viral abundances increase encounters, and are predicted to increase lytic production in the simplest possible scenario where all other infection variables are maintained. However, the rise of defense, lysogeny, or other unknown mechanism can prevent the increase in viral abundances. Al-Rasheed et al.^5^ criticized our interpretation that lysogeny may be the underlying mechanism, and certainly, lysogeny is not the only possible explanation, as acknowledged in our original publication.

We also acknowledge that computational strategies to infer phage–host associations are currently limited, and curtail the ability to observe associations between phage–host pairs^8–10^. Nevertheless, we adopted the best strategies available to infer these associations. As computational tools improve, so will the capacity to re-evaluate the validity of the phage–host abundance patterns presented in our original publication.

Other independent studies have reported findings that corroborate the Piggyback-the-Winner model. The recently discovered Arbitrium system^11,12^ demonstrated experimentally not only that phages can communicate to promote a shift to the lysogenic life style when encounter rates are high, but also described the molecular mechanism that controls this switch. Second, comparative genomics demonstrated that prophages are more prevalent among the genomes of organisms with higher growth rates^13^. Last, metagenomic data obtained from the murine gut, an ecosystem with high host abundances, has demonstrated that lysogeny is the preferred strategy adopted by phages in this habitat^14^.

Finally, in Coutinho et al.^1^ we performed no direct measurement of lytic production or frequency of lysogeny, nor did we claim to do so. We utilized the plethora of publicly available metagenomic and viromic data to gain insight on replication strategies. Al-Rasheed et al.’s^5^ analyses do not contradict the core interpretation of relatively lower lytic activity with increasing host abundance. In our original discussion^1^, we presented rationale supporting PtW as the underlying mechanism. But more importantly, we encourage further investigation of viral life strategies in the light of new hypotheses and data.
